# Proliferation of measures contributes to advancing psychological science

**DOI:** 10.1038/s44271-024-00065-w

**Published:** 2024-03-09

**Authors:** Dragos Iliescu, Samuel Greiff, Matthias Ziegler, Christopher Nye, Kurt Geisinger, Martin Sellbom, Douglas Samuel, Donald Saklofske

**Affiliations:** 1 University of Bucharest, Bucharest, Romania; 2 Stellenbosch University, Stellenbosch, South Africa; 3 Luxembourg University, Esch-sur-Alzette, Luxembourg; 4 Humboldt-Universität, Berlin, Germany; 5 Michigan State University, East Lansing, MI USA; 6 University of Nebraska Lincoln, Lincoln, NE USA; 7 University of Otago, Dunedin, New Zealand; 8 Purdue University, West Lafayette, IN USA; 9 Western University, London, Canada

**Keywords:** Human behaviour, Human behaviour

## Abstract

Proliferation and variability of psychological measures are part of the scientific process. While sometimes an indication of questionable research practices, there are also benign reasons for measurement proliferation and the community’s response must take both aspects into account.

## Teaser

Proliferation and variability of psychological measures are part of the natural workings of the scientific process. They contribute to theory development, transparency, replicability, and validity, and restricting proliferation might hinder progress in the psychological sciences.

## Introduction

It is old news that psychology is going through a serious replication and credibility crisis. In searching for solutions, several phenomena have been pointed out as potential causes^1^: overemphasis on statistical significance, publication bias, inadequate statistical power, weak specification of theories and analysis plans, etc. A currently much-debated issue is the proliferation and variability of measures that are typically found in psychological assessment^2^. The scientific community is concerned that such proliferation may lead to questionable measurement practices^3^ and has therefore recommended guidelines to counter the proliferation of trivial and redundant measures^4^. Such guidelines suggest that we should, for example, aspire to demonstrate non-redundancy, report and justify modifications in scales, and provide evidence on different sources of validity (including incremental validity) for any new or modified instrument. Following these guidelines may alleviate the phenomenon to some extent, but we expect and support the proliferation of psychological measures to continue because of its relevance for theory development and validation.

Here, we advance that psychological assessment is an important force in the current trend of replicability, robustness, and reproducibility of psychological science^1^, discuss reasons why desired theoretical and empirical advances in psychological assessment will rather lead to a proliferation and not a restriction of measures, and outline some of the positive outcomes of such proliferation. By doing so, we suggest that proliferation of measures is not *per se* a negative phenomenon, but strongly depends on how it is situated and that it can be bound into the very fabric of how psychological science develops.

There are many reasons for the proliferation and variability of psychological measures, and while the phenomenon may be frustrating at times, many of these reasons are logical and defensible. We elaborate more on two of the benign reasons for measurement proliferation.

### Measures are context dependent

The validity of any score derived from a psychological measure is based on how well the underlying measure is aligned with the specific context in which it is used. The context of any study is multidimensional. It involves, amongst others, characteristics of the audience (e.g., language, culture, age) and of instrumentation (e.g., administration procedure). Context is a powerful force when researchers prepare their instruments, motivating them to change wording, adapt materials, shorten the item pool, or otherwise modify the original forms of tests. These types of (minor) adaptations are often necessary for maintaining the validity of a measure in a specific context and are not limited to the rather well-documented domain of linguistic/cultural test adaptation^3^.

We agree that contextual adaptations require both judgmental and empirical evidence to justify their use, as suggested in several guidelines and test standards^3,4^. On the other hand, the justifications required also need to be embedded into existing practicalities such as the availability of samples, time constraints, and others: few studies are able to examine all possible sources of validity evidence needed for contextual changes and most researchers have to find a balance between the a-priori need for instrument validity and the specific demands of the study—or not conduct the study at all.

Thus, we argue that proliferation and variability, when sufficiently supported by empirical evidence and when made in the spirit of aligning a measure with the specific study context, are reasonable. Cronbach himself^5^, near the end of his long career and some 34 years after he and Paul Meehl called for construct validation, argued that nomological networks and the logical positivism that underlies them would not give justice to the complexity and changing nature of the world.

### Measures with the same label may tap into different constructs

The relation between psychological constructs and specific measures is anything but isomorphic and a direct mapping between a particular psychological construct and a specific measure is usually difficult or impossible to achieve as has been shown across subfields of psychology. In many cases, there are multiple underlying theories behind the same construct. Psychopathy is a good example: the proliferation of psychopathy measures matches the proliferation of theories and perspectives of this construct^6^, and several measures are needed to adequately map onto all theoretical perspectives. This issue was foreseen by Cronbach and Meehl^7^ in their definition of the nomological net. While they delineated the importance of defining relations between constructs and manifest variables, they also acknowledged that theories about constructs might evolve or be developed based on empirical findings, necessitating new measures to assess the revised constructs. The idea of a repository for measures and data was suggested as a logical evolution of this line of thinking^2,8^.

Thus, we argue that the proliferation and variability of psychological measures are a direct result of the scientific process. As theoretical ideas are refined, new populations come into focus, or the number of use cases increases, new measures are developed and most of them will be either quickly dismissed or remain reserved for specific purposes, but some of them will experience wide use. This process leads not to fewer but to more (and eventually higher-quality) measures. Meaningful latent constructs converge and emerge through such a proliferation of measures. The entire replicability and transparency movement relies heavily on this idea: relations between theoretical constructs are generalizable only as far as they stand the test of diversity and are confirmed in different samples, with variable measures, and across different contexts. Instead of being considered negative, this observed fragmentation of measures within theoretical constructs can be interpreted as the continuous development of psychological science, if certain quality-ensuring steps are adhered to.

## What would be the consequences of less measurement proliferation?

Unjustified variability in psychological measures should be discouraged—but it is difficult to ascertain when variability is indeed meaningless. At the same time, programmatic stifling of such variability is likely to be detrimental if taken to the extreme and blindly imposed or enforced across situations. This was the case, for instance, when large funders of mental health research around the world announced in 2020 their plans to standardize mental health measurement^9^. The recent SOBER guidelines^2^, which also attempt to inhibit such variability are well-intentioned but may have little impact if not enforced by funders and journals.

### Standardization may decrease the validity

A nuanced understanding of psychological constructs implies that there are different ways of measuring the same construct and that adaptations are not a threat to (construct) validity but an empirical test of it. For example, tests of general mental ability (GMA) show a high level of convergence, which has firmly established their construct validity: it is exactly the proliferation of measures that have furthered our understanding of GMA. In fact, one could argue that there are few (if any) psychological constructs that exhibit as much measurement proliferation as GMA showing that sometimes theory and validity development go hand in hand with the proliferation of measures.

### Lack of variability may decrease self-correction and theory development

Science progresses, in psychology as in other fields, through diversity. Competing theories appear and are pitted against each other, and measurement approaches or new instruments come as a companion to these theoretical advances. Measures, just like theories, survive or die when confronted with each other: researchers and practitioners tend to not use outdated or poorly performing measures when better ones are available. In this regard, science is self-corrective: for example, test-related systematic reviews and good practice guidelines^10^ help in relation to test revisions, obsolete tests, and test disposal.

### Standardization may hamper replication and theory validation

Restricting variability in psychological measures might prove detrimental to replication efforts. We believe that minor adaptations in psychological measures should not lead to radically different results: in those cases where even minor adaptations in a psychological measure lead to (substantially) different results, it is reasonable to question the robustness of the initial findings on the latent level. Conceptual replications, in fact, require alternative measures of the same construct^1^. Just as the diversity of different populations included in psychological research (e.g., beyond WEIRD) increases generalizability, the same holds for the diversity of psychological measures.

## Conclusion

We have argued, in line with extant discussions in the scientific community, that the proliferation of measures may have positive effects and that we need a better understanding of both the underlying reasons for the proliferation of psychological measures and its consequences before restricting measurement proliferation. Simply focusing on reducing variation in measures, without any consideration of the benign and justifiable reasons for this variation, can backfire and may lead to slower theory development, less transparency, and lower validity. Existing guidelines recognize that uniform policies for test development and evaluation may not apply in all situations and, therefore, take a non-prescriptive stance while still providing comprehensive guidance on state-of-the-art test development practices.

We believe that understanding and embracing the fact that measure proliferation is part of how psychology evolves, is the first step towards making good use of this phenomenon. We advance that the solution is not to stifle this force but to use it, by (more) openly sharing information about any and all measures. However, we believe that good intentions regarding data sharing may not be sufficient for a systemic change, and we suggest that the change should be actively driven—for example by elaborating in more detail the technical standards for such (possibly automated) exchange of information.

The tentative suggestion of an open repository was made^2^, containing “measurement protocols” with machine-readable metadata that are analyzable through large language models. Establishing such a repository for (adapted) measures and data obtained with them is a worthwhile endeavor, both in the context of the more recent calls for transparency and reproducibility, and of the classical calls for continuous scrutiny of nomological networks of psychological constructs. If implemented, such a repository will likely advance research into how measures relate to each other and to their focal constructs. However, in order for such an initiative to be successful, we believe that the crucial element is the development of a technical standard for test meta-data. Such a standard for possibly automated exchange of information on test data could be developed through collaborative work by an international expert task force.

### Supplementary information

Peer Review File
