# Supplementary material for: Proliferation of measures contributes to advancing psychological science
Source: Commun Psychol. 2024 Mar 9;2:19. doi: 10.1038/s44271-024-00065-w (PMC11332101; doi:10.1038/s44271-024-00065-w)
Supplement: Supplementary file 1 — Peer Review File [file 44271_2024_65_MOESM1_ESM.pdf]

nature portfolio

Peer Review File

Proliferation of measures contributes to advancing psychological science

Open Access This file is licensed under a Creative Commons Attribution 4.0 International License, which permits use, sharing, adaptation, distribution and reproduction in any medium or format, as long as you give appropriate credit to the original author(s) and the source, provide a link to the Creative Commons license, and indicate if changes were made. In the cases where the authors are anonymous, such as is the case for the reports of anonymous peer reviewers, author attribution should be to 'Anonymous Referee' followed by a clear attribution to the source work. The images or other third party material in this file are included in the article's Creative Commons license, unless indicated otherwise in a credit line to the material. If material is not included in the article's Creative Commons license and your intended use is not permitted by statutory regulation or exceeds the permitted use, you will need to obtain permission directly from the copyright holder. To view a copy of this license, visit http://creativecommons.org/licenses/by/4.0/.

Web links to the author’s journal account have been redacted from the decision letters as indicated to maintain confidentiality

9th Nov 23

Dear Dragos,

Thank you for submitting your manuscript titled "Brushing harder does not clean better. On toothbrushes and measurement proliferation in psychology: A reply to Elson et al. (2023)" to Communications Psychology.

I have read your manuscript with a lot of interest and in principle, we would be happy to take it forward to peer-review. However, due to certain shortcomings I am concerned that sending the current manuscript out to review could lead to unnecessary delays and quite possibly an undesirable outcome of the review process.

In particular, your manuscript is presently written as a direct reply to a recent publication in Communications Psychology (Elson, M., Hussey, I., Alsalti, T. et al. Psychological measures aren't toothbrushes. Commun Psychol 1, 25 (2023)). We do not publish direct reply formats for opinion content, which means that as written, the piece fits poorly with journal requirements. You make a list of points that I believe will be of interest to our readers, regardless of whether they have read Elson et al. In other words, your contribution can be peer-reviewed as a standalone piece, in the Comment format.

We appreciate the importance of debate, and you are naturally welcome to criticize existing pieces in our pages. However, to ensure your piece is in itself of broad interest to readers in psychology, not only those who have read Elson et al., you would need to revise the text to make it less about another publication, and focus more strongly on your own message. You may of course continue cite Elson et al., and pinpoint the faultlines of the respective arguments.

I would therefore like to invite you to revise your manuscript to address these concerns before we make a final determination on whether to send your manuscript for external review.

I shall hope to receive your revised version as soon as you are able to complete the suggested revisions. If something similar is published in the interim we will have to consider the impact it has on the novelty of a revised manuscript.

If you anticipate a delay of more than four weeks, please let us know. Should your manuscript be substantially delayed without notifying us in advance and your article is eventually published, the received date may be that of the revised, not the original, version.

If you are not interested in submitting a suitably revised manuscript in the future please let me know immediately so we can close your file. If you have any questions, including specific questions regarding the requested revisions, please contact me.

Please use the link below when you are prepared to resubmit.
[link redacted]

Thank you for your interest in Communications Psychology.

Best wishes,
Marike

Marike Schiffer, PhD
Chief Editor
Communications Psychology

20th Dec 23

Dear Dragos,

Your Comment titled "The proliferation of measures in psychology: Between frustration and need" has now been seen by 2 referees, whose comments appear below. In light of their advice, I am delighted to say that we are happy, in principle, to publish it in Communications Psychology under a Creative Commons 'CC BY' open access license.

You will see that the referees provided critical, constructive comments. We ask you to engage thoroughly with their concerns, and I will attach an edited version of your manuscript to explain where we see the greatest need for revisions. Note that you are not required to provide any data or analyses - Comments should be pure opinion pieces, not research articles. Data are always considered anecdotal in this format and only allowed in cases where they are strictly relevant to the argument, which does not apply to your case.

We will not send your revised paper for further review if, in the editors' judgment, the referees' comments on the present version have been addressed. If the revised paper is in Communications Psychology format, in an accessible style, and of appropriate length, we shall accept it for publication immediately. I have attached an edited version of you manuscript, and ask you to attend to each comment in detail.

EDITORIAL REQUESTS:

* Please review the changes in the attached copy of your manuscript, which has been edited for style, and address the comments and queries I have added. If using Word, please use the 'track changes' feature to make the process of accepting your manuscript more efficient.

* Communications Psychology uses a transparent peer review system. On author request, confidential information and data can be removed from the published reviewer reports and rebuttal letters prior to publication. If you are concerned about the release of confidential data, please let us know specifically what information you would like to have removed. Please note that we cannot incorporate redactions for any other reasons.

*If you have not done so already, please alert me to any related manuscripts from your group that are under consideration or in press at other journals, or are being written up for submission to other journals (see www.nature.com/authors/editorial_policies/duplicate.html for details).

FORMATTING GUIDELINES:
You will find a complete list of formatting requirements following this link:
https://www.nature.com/documents/commsj-style-formatting-checklist-comment.pdf
Please use the checklist to prepare your manuscript for final submission. In the following, I also highlight some issues of particular importance.

** Title
Titles should be descriptive of the main message your manuscript conveys and should not exceed 90 characters (including spaces). Please note that punctuation is not allowed, nor are titles of the following format: "title: subtitle".

** Preface
The paper's preface (up to 40 words; without references) should serve both as a general introduction to the topic, and highlight your position or proposal. Because we hope that researchers across all fields of psychology will be interested in your work, the preface should be as accessible as possible.

** Length
The ideal length for Comment article in Communications Psychology is 1,500 words. We have some flexibility, however, but please ensure that your text does not exceed 1,800 words.

** Main text

Please provide three or four section headings in the main text. These should relate to the content of the article rather than being generic. Headings should be no longer than 35 characters (including spaces) and should not use punctuation.

* References
References appear as superscript Arabic numerals, in order of mention. The reference list mentions references in the numerical order in which they are mentioned in the main text. If a reference is cited more than once, the same number is used throughout the text and the reference receives a single entry in the reference list.

Only papers that have been published or accepted by a named publication should be in the reference list (preprints and citations of datasets are also permitted). Unpublished/Submitted research should not be included in the reference list; it should only be mentioned briefly and parenthetically in the main text. Note that no major arguments should rely on unpublished research.

Published conference abstracts and URLs for websites should be cited parenthetically in the text, not in the reference list.

Footnotes are not used.

* Competing interests
Please include a "Competing interests" statement after the References. Note that we ask authors to declare both financial and non-financial competing interests. For more details, see https://www.nature.com/authors/policies/competing.html. If you have no financial or non-financial competing interests, please state so: “The authors declare no competing interests.”

SUBMISSION INFORMATION:

In order to accept your paper, we require the following:

* A cover letter describing your response to our editorial requests.

* The final version of your text as a Word or TeX/LaTeX file, with any tables prepared using the Table menu in Word or the table environment in TeX/LaTeX and using the 'track changes' feature in Word.

* OPTIONAL: A separate document detailing your point-by-point response to any issues raised by our referees (please include the referees' comments in this document).

At acceptance, the corresponding author will be required to complete an Open Access Licence to Publish on behalf of all authors, declare that all required third-party permissions have been obtained.

Please note that your paper cannot be sent for typesetting to our production team until we have received this information; **therefore, please ensure that you have this ready when submitting the final version of your manuscript.**

ORCID

Communications Psychology is committed to improving transparency in authorship. As part of our efforts in this direction, we are now requesting that all authors identified as ‘corresponding author’ create and link their Open Researcher and Contributor Identifier (ORCID) with their account on the Manuscript Tracking System (MTS) prior to acceptance. ORCID helps the scientific community achieve unambiguous attribution of all scholarly contributions. For more information please visit http://www.springernature.com/orcid

For all corresponding authors listed on the manuscript, please follow the instructions in the link below to link your ORCID to your account on our MTS before submitting the final version of the manuscript. If you do not yet have an ORCID you will be able to create one in minutes.

https://www.springernature.com/gp/researchers/orcid/orcid-for-nature-research

IMPORTANT: All authors identified as ‘corresponding author’ on the manuscript must follow these instructions. Non-corresponding authors do not have to link their ORCIDs but are encouraged to do so. Please note that it will not be possible to add/modify ORCIDs at proof. Thus, if they wish to have their ORCID added to the paper they must also follow the above procedure prior to acceptance.

To support ORCID's aims, we only allow a single ORCID identifier to be attached to one account. If you have any issues attaching an ORCID identifier to your MTS account, please contact the Platform Support Helpdesk.

Please use the following link to submit the above items:
[link redacted]** This url links to your confidential home page and associated information about

manuscripts you may have submitted or be reviewing for us. If you wish to forward this email to co-authors, please delete the link to your homepage first **

We hope to hear from you within four weeks; please let us know if the process may take longer.

Best wishes,

Marike

Marike Schiffer, PhD
Chief Editor
Communications Psychology

REVIEWERS' EXPERTISE:

Reviewer #1: measurement, reproducibility
Reviewer #2: measurement, reproducibility

REVIEWERS' COMMENTS:

Reviewer #1 (Remarks to the Author):

Mixed feelings about this potential contribution: although comment on original work is, of course, always welcome, the original work presented large-scale data for supporting the views, suggestions, and proposals of the original authors, whilst this submitted commentary does not present any further data. Hence, it constitutes an opinion piece throughout, and I found it difficult to agree with the authors' various claims, starting from the statement in the opening paragraph that the suggested guidelines are "prescriptive" and would be "artificially restricting". Again, line 77: "viewed by us as overly restrictive". My understanding was and is that these guidelines currently constitute a grassroots and bottom-up voluntary proposal, i.e., you and me and others CAN use it as a guidance - so why "overly restrictive"?

Lines 39-40: the example presented here, i.e., the RSES (Rosenberg Self-Esteem Scale) in my view actually is a counter-example, because self-esteem is a quite broad concept, but the RSES has been very widely used and, in fact, is one of the most widely used (and thus most widely cited) self-report instruments across psychological science. Regarding the statement that the RSES item pool should be rephrased "when used in a sample of school children, and if used in a sample of immigrants": I wondered whether any such contextualizing, very specific, adaptations of the RSES are out there (the authors do not cite any) – this claim might perhaps be more a form of social constructivism to which psychometricians would not agree.

Also, some strawman argumentation seems to be put forward: RE "a single measure" of psychopathy – this is not the case, and I think it has not been argued for.

I also have to disagree with the claim that "proliferation and variability of psychological measures are a direct result of the scientific process, and this process leads not to fewer but to more (and

higher-quality) measures". Why should this exactly be the case? One would surmise that progress eventually leads to weeding out of the less than optimal measures. What is absent in this argumentation is the key concept of incremental validity in psychological assessment and scale development and validation: an alternative, novel measures must show superiority relative to existing measures. This key concept of incremental validity as one important, informative facet of the validation framework is glaringly absent in this text.

RE "fragmentation can be interpreted as the continuous development of psychological science": I have to disagree here as well. Same argumentation as above: think, for instance, of the phenomena of consilience, universality, generalizability across and within science fields. From an epistemic point of view, fragmentation of concepts and knowledge can hardly be a sign of scientific progress. This debatable argument reappears throughout the text, e.g., line 87: "science progresses … through diversity".

Another claim is (one again, presented without any refs) that "even minor adaptations in a psychological measure lead to (substantially) different results". Is this really the case, and would this be generalizable? Think of parallel test forms; they usually work quite well.

Line 108, "(more) openly sharing information about any and all measures". This was already included in the SOBER guidelines.

The authors end on the note that "a non-prescriptive stance" should be taken, but fail to elaborate on through which forms and means this could precisely be achieved (i.e., no alternative proposal is outlined).

Minor comment: the opening paragraph is entitled "Preface". This is a little bit unusual.

Reviewer #2 (Remarks to the Author):

Thank you for the invitation to review 'The proliferation of measures in psychology: Between frustration and need' for Communications Psychology.

The commentary is fine as is, but could be strengthened further. I provide comments below.

Note that we have written about similar measurement issues, sometimes based on the exact arguments and rationale the authors here provide. It feels like the authors could strengthen the work by referring to some of this. On the one hand, I understand this is unusual and often a red flag, so I leave it to the authors and editor to decide whether some of these references would be helpful. On the other, I assume I was invited to review this paper because we have done a lot of work on the topic, and it would be remiss not to point out potential work on the topic.

Broadly, the authors respond to a piece with the argument that there are 2 justifiable reasons for measure proliferation.

(1) Summarized: "Different measures for construct X may depend on the circumstance/situation, i.e. measures are on text dependent (e.g. language, population)."

I think you could rephrase the header, most readers will have a hard time understanding that. Some of the language seems unnecessary ("in virtually all cases idiosyncratic"), there are of course cross-cultural or cross-setting studies less idiosyncratic than others. We have written about context in some detail in our piece arguing against mandating a particular depression instrument by NIH/WELLCOME that seems relevant in this context: https://pubmed.ncbi.nlm.nih.gov/32986859/. Given that the authors of the original piece the authors comment on will agree that different language versions should exist, this does not count as proliferation, and I would look for stronger examples the authors may disagree with.

(2) Summarized: "Different measures for construct X may tap into different constructs X"
We talk about this in detail in here: https://www.nature.com/articles/s44159-022-00050-2, discussing proliferation of depression measures and how they were developed for different contexts and may tap into different constructs. An issue I see with the line of reasoning the authors provide is that psych scales rarely start with a clear construct definition and then properly measure that—this is also the case when interrogating the scales in our work. They were developed for a purpose (e.g., prevalence rate estimation vs tracking treatment progress), but clear (in the best case formal) theories of constructs are absent, making it difficult to evaluate whether measures measure the purported constructs because those weren't clearly defined. In that sense, one could disagree with the authors that proliferation "leads to more (higher quality" scales. This is only the case if constructs are clearly defined, which isn't the case. From our overview on the topic (https://journals.sagepub.com/doi/10.1177/2515245920952393):
"Barry, Chaney, Piazza-Gardner, and Chavarria (2014) reported that between 40% and 93% of measures used across seven journals in educational behavior lacked validity evidence, and Weidman, Steckler, and Tracy (2017) reported that among the 356 measurement instances coded in their review of emotion research, 69% included no reference to prior research or a systematic development process. In their review of the relation between technology use and well-being, Orben and Przybylski (2019) reported that researchers pick and choose within and between questionnaires, "making the pre-specified constructs more of an accessory for publication than a guide for analyses" (p. 181).

The authors then continue to look into consequences of less proliferation.
- "Unjustified variability in psychological measures should be discouraged – but it is difficult to ascertain when variability is indeed meaningless". I think there is decent literature on this—would anybody consider it reasonable to have 280 depression scales?
https://www.tandfonline.com/doi/abs/10.1207/s15366359mea0403_1
- "At the same time, programmatic stifling of such 74 variability is likely to be detrimental if taken to the extreme and blindly imposed or enforced across situations". Agreed, and there is literature on this, including a number of arguments: https://pubmed.ncbi.nlm.nih.gov/32986859/

Best of luck with the manuscript!
To improve the transparency and accountability of the peer review process, I sign my reviews
Eiko Fried, Leiden University

Dear Editor,

We would like to thank you and the reviewers very much for taking the time to carefully read and comment on our submission to Communications Psychology with reference COMMSPSYCHOL-23-0377A.

We appreciate the valuable feedback and the opportunity to resubmit the manuscript with modifications. We respond below to reviewers' comments and also highlight changes that we implemented in the text. In this, we have gladly implemented the suggestions of the reviewers and hope that the manuscript is now eligible for publication in Communications Psychology.

Sincerely yours,

***

Reviewer #1 (Remarks to the Author):

Mixed feelings about this potential contribution: although comment on original work is, of course, always welcome, the original work presented large-scale data for supporting the views, suggestions, and proposals of the original authors, whilst this submitted commentary does not present any further data. Hence, it constitutes an opinion piece throughout, and I found it difficult to agree with the authors' various claims, starting from the statement in the opening paragraph that the suggested guidelines are "prescriptive" and would be "artificially restricting".

>> Authors' response: Thank you for your comments. We have used them to refine the wording and, we believe, the fundamental tone of the paper. To the opening paragraph that you referred to: We have now changed the invoked stance in the preface, and have deleted both the word "artificially" and the suggestion that some guidelines may be "prescriptive".

Again, line 77: "viewed by us as overly restrictive". My understanding was and is that these guidelines currently constitute a grassroots and bottom-up voluntary proposal, i.e., you and me and others CAN use it as a guidance -so why "overly restrictive"?

>> Authors' response: Thank you very much for this important comment. We agree with your comment that, at this point in time, the guidelines that we refer to constitute a bottom-up voluntary proposal and have not seen broad implementation yet. In our commentary, we don't argue that their current impact has created a factually overly restrictive situation when it comes to developing new psychological measures. Instead, we highlight that the purpose of such guidelines is to seek comprehensive implementation across the field and that, through this, these guidelines have the potential to alter the landscape of the field. On this background, we argue that the content of the guidelines – even though currently at the "grass-root" level – can be perceived as overly restrictive and require careful reconsideration before being put into practice. Our core argument is that the consequences of such guidelines might not have been sufficiently appreciated, in particular with regard to unintended side effects and that a careful process of weighing the pros and cons of measurement proliferation need to be reflected in any new guideline. In our view, such a process was not sufficiently implemented in recent guidelines, which motivated us to write this commentary with a broad allusion to the question of measurement proliferation in psychology. In the revised version, we have adapted the sentence on line 77 that you highlight in your comment. We have deleted the wording "viewed by us as overly restrictive". We have also introduced another, stronger example, and have rephrased reference to SOBER, i.e.,: "The recent SOBER guidelines, which also attempt to inhibit such variability are well-intentioned, but may have little impact if not enforced by funders and journals".

Lines 39-40: the example presented here, i.e., the RSES (Rosenberg Self-Esteem Scale) in my view actually is a counter-example, because self-esteem is a quite broad concept, but the RSES has been very

widely used and, in fact, is one of the most widely used (and thus most widely cited) self-report instruments across psychological science. Regarding the statement that the RSES item pool should be rephrased "when used in a sample of school children, and if used in a sample of immigrants": I wondered whether any such contextualizing, very specific, adaptations of the RSES are out there (the authors do not cite any) – this claim might perhaps be more a form of social constructivism to which psychometricians would not agree.

>> Authors' response: Thank you very much for flagging some relevant inconsistency in the example of the RSES. After careful consideration and with the length restrictions for commentaries in mind (the revised version is about 300 words longer than commentaries usually are), we have decided to delete the example entirely as it is not essential for our argument and we are confident that our argument will be equally accessible to readers even without the example.

Also, some strawman argumentation seems to be put forward: RE "a single measure" of psychopathy – this is not the case, and I think it has not been argued for.

>> Authors' response: Thank you for your comment. We did not intend this to be a strawman argumentation – our line of argumentation was to suggest that there is a fine line after which restriction of measures becomes ridiculous. But we see now how this could be misconstrued, and have now changed the text to "… and several measures are needed in order to map onto all theoretical perspectives".

I also have to disagree with the claim that "proliferation and variability of psychological measures are a direct result of the scientific process, and this process leads not to fewer but to more (and higher-quality) measures". Why should this exactly be the case? One would surmise that progress eventually leads to weeding out of the less than optimal measures. What is absent in this argumentation is the key concept of incremental validity in psychological assessment and scale development and validation: an alternative, novel measures must show superiority relative to existing measures. This key concept of incremental validity as one important, informative facet of the validation framework is glaringly absent in this text.

>> Authors' response: Thank you for your comment and to flagging this concern. We have now redesigned the corresponding paragraph to explain our reasoning with more care. In our revision, we have considered in this paragraph also your next note (see immediately below) and we describe there in more detail how we proceeded with the revision.

RE "fragmentation can be interpreted as the continuous development of psychological science": I have to disagree here as well. Same argumentation as above: think, for instance, of the phenomena of consilience, universality, generalizability across and within science fields. From an epistemic point of view, fragmentation of concepts and knowledge can hardly be a sign of scientific progress. This debatable argument reappears throughout the text, e.g., line 87: "science progresses … through diversity".

>> Authors' response: Thank you for flagging your concern with regard to fragmentation in psychological science and the argument that we put forward. We believe that there might be a misunderstanding here. In our argument, we do not advocate for fragmentation of theoretical concepts in science (and we agree with the reviewer that we have too much of this across psychology). Our commentary is related to the proliferation of psychological _measures_ within theoretical concepts and we argue that, as theoretical ideas are refined and extended, new populations become the focus of investigation, or the number of use cases increases, proliferation of measures or fragmentation of measures is part of this process as all these different contextual factors might lead to (slightly) modified measures. Most of them will be either quickly dismissed or remain reserved for specific purposes, but some of them will experience wide use – this is the pattern that is to be expected and that is also highly consistent with the pattern reported in the recently published commentary to which we reference: Most measures are not used often and a minority experience wide use. Having multiple measures available

actually provides an opportunity to test whether fragmented measures (as these slightly modified versions could be called) converge on an empirical and conceptual level and this serves as one of the strongest possible tests for the robustness of the underlying theoretical concept.

In the revised version of the paper, we have tried to clarify our argument and to avoid any potential for misunderstanding. It now reads: “Thus, we argue that proliferation and variability of psychological measures are a direct result of the scientific process. As theoretical ideas are refined, new populations come into focus, or the number of use cases increases, new measures are developed and most of them will be either quickly dismissed or remain reserved for specific purposes, but some of them will experience wide use. This process leads not to fewer but to more (and eventually higher-quality) measures. Meaningful latent constructs converge and emerge through such proliferation of measures. The entire replicability and transparency movement relies heavily on this idea: relations between theoretical constructs are generalizable only as far as they stand the test of diversity and are confirmed in different samples, with variable measures, and across different contexts. Instead of being considered negative, this observed fragmentation of measures within theoretical constructs can be interpreted as the continuous development of psychological science, if certain quality ensuring steps are adhered to.”

Another claim is (one again, presented without any refs) that “even minor adaptations in a psychological measure lead to (substantially) different results”. Is this really the case, and would this be generalizable? Think of parallel test forms; they usually work quite well.

>> Authors’ response: Thank you again for challenging our ideas. The point we intended to make here was that minor adaptations in a psychological measure should not lead to substantially different results on the conceptual level and, if they do, one might question the robustness of the theoretical construct. Along this line of thinking, proliferation of measures can help to test conceptual robustness and generalizability. We did not suggest that minor adaptations lead to different result, but intended the text as an “what if” case: if even minor adaptations would lead to different results, then the initial findings would really be questionable. We have now revised our manuscript to add clarity. The revised text now reads: “We believe that minor adaptations in psychological measures should not lead to radically different results: in those cases where even minor adaptations in a psychological measure lead to (substantially) different results, it is reasonable to question the robustness of the initial findings on the latent level.”

Line 108, “(more) openly sharing information about any and all measures”. This was already included in the SOBER guidelines.

>> Authors’ response: We do not contradict the meaningfulness of this proposal at all. We only think that it’s too soft and more decisive action could be taken, building on technology. We have added this sentence: “However, we believe that good intentions regarding data sharing may not be sufficient for a systemic change, and we suggest that the change should be actively driven – for example by elaborating in more details the technical standards for such (possibly automated) exchange of information.”

The authors end on the note that “a non-prescriptive stance” should be taken, but fail to elaborate on through which forms and means this could precisely be achieved (i.e., no alternative proposal is outlined).

>> Authors’ response: Thank you for challenging us to rethink our Conclusion section. We have now reorganized the three paragraphs in our Conclusions.

We have both shifted some of the text around, and have added new text. The flow of the Conclusion section is now thus: (1) We formulate a summary of our ideas and point out that there may be benign and justifiable reasons for variation in measures. (2) We point out that data sharing will exploit in a positive way, to the benefit of scientific progress, this tendency towards proliferation – but that good

intentions are not enough. (3) We drive the proposal of a repository of measurement protocols but suggest that technical standards for automatization need to be developed.

Minor comment: the opening paragraph is entitled “Preface”. This is a little bit unusual.

>> Authors’ response: Thank you so much. We have deleted the title “preface” in the revised version of the manuscript.

Reviewer #2 (Remarks to the Author):

Thank you for the invitation to review ‘The proliferation of measures in psychology: Between frustration and need’ for Communications Psychology.

The commentary is fine as is, but could be strengthened further. I provide comments below.

Note that we have written about similar measurement issues, sometimes based on the exact arguments and rationale the authors here provide. It feels like the authors could strengthen the work by referring to some of this. On the one hand, I understand this is unusual and often a red flag, so I leave it to the authors and editor to decide whether some of these references would be helpful. On the other, I assume I was invited to review this paper because we have done a lot of work on the topic, and it would be remiss not to point out potential work on the topic.

Broadly, the authors respond to a piece with the argument that there are 2 justifiable reasons for measure proliferation.

(1) Summarized: “Different measures for construct X may depend on the circumstance/situation, i.e. measures are context dependent (e.g. language, population).”
I think you could rephrase the header, most readers will have a hard time understanding that. Some of the language seems unnecessary (“in virtually all cases idiosyncratic”), there are of course cross-cultural or cross-setting studies less idiosyncratic than others. We have written about context in some detail in our piece arguing against mandating a particular depression instrument by NIH/WELLCOME that seems relevant in this context: https://pubmed.ncbi.nlm.nih.gov/32986859/. Given that the authors of the original piece the authors comment on will agree that different language versions should exist, **this does not count as proliferation, and I would look for stronger examples the authors may disagree with.**

>> Authors’ response: Thank you for this important comment. We have changed the heading as you advised. The heading in the revised version of the manuscript now reads “measures are context dependent”. We have reworded the text pointed out by you by deleting “and in virtually all cases highly idiosyncratic”. We have also added a half-sentence clarifying that our argument do not relate to different language versions and that this would not be contested in the SOBER guidelines. The sentence at the end of the paragraph now reads: “…and are not limited to the rather well documented domain of linguistic/cultural test adaptation.” Thank you also for pointing us here and elsewhere in your review to important references and examples that are relevant to the content of our commentary. With regard to specific examples, we decided to cut down on them in general across the revised version of the manuscript because (1) Reviewer 1 did not agree with several of them and we realized that there is quite some potential for misunderstanding when using examples without extensively elaborating on their specific context and meaning and (2) we are aware that we need to keep the length of this commentary within reasonable limits (it is already overlength now). However, we did manage to add one of the references that you provided in the revised version of the manuscript.

(2) Summarized: “Different measures for construct X may tap into different constructs X”
We talk about this in detail in here: https://www.nature.com/articles/s44159-022-00050-2, discussing

proliferation of depression measures and how they were developed for different contexts and may tap into different constructs. An issue I see with the line of reasoning the authors provide is that psych scales rarely start with a clear construct definition and then properly measure that—this is also the case when interrogating the scales in our work. They were developed for a purpose (e.g., prevalence rate estimation vs tracking treatment progress), but clear (in the best case formal) theories of constructs are absent, making it difficult to evaluate whether measures measure the purported constructs because those weren't clearly defined. In that sense, one could disagree with the authors that proliferation "leads to more (higher quality" scales. This is only the case if constructs are clearly defined, which isn't the case. From our overview on the topic (https://journals.sagepub.com/doi/10.1177/2515245920952393): "Barry, Chaney, Piazza-Gardner, and Chavarria (2014) reported that between 40% and 93% of measures used across seven journals in educational behavior lacked validity evidence, and Weidman, Steckler, and Tracy (2017) reported that among the 356 measurement instances coded in their review of emotion research, 69% included no reference to prior research or a systematic development process. In their review of the relation between technology use and well-being, Orben and Przybylski (2019) reported that researchers pick and choose within and between questionnaires, "making the pre-specified constructs more of an accessory for publication than a guide for analyses" (p. 181).

>> Authors' response: Thank you for mentioning these important points and for embedding our commentary within the broader scope of the discussion on valid measurement in psychological science. We wish we could embark on a more comprehensive appreciation of the arguments (and the literature) that you provide within our commentary but the nature of such a commentary does not allow for an in-depth discussion of all of these important points. However, we tried to allude to some of them throughout the manuscript and in the revised version we also cite one of the references that you provided (keeping the strict limit of 10 references for commentaries in mind).

The authors then continue to look into consequences of less proliferation.
- "Unjustified variability in psychological measures should be discouraged – but it is difficult to ascertain when variability is indeed meaningless". I think there is decent literature on this—would anybody consider it reasonable to have 280 depression scales? https://www.tandfonline.com/doi/abs/10.1207/s15366359mea0403_1
- "At the same time, programmatic stifling of such variability is likely to be detrimental if taken to the extreme and blindly imposed or enforced across situations". Agreed, and there is literature on this, including a number of arguments: https://pubmed.ncbi.nlm.nih.gov/32986859/

>> Authors' response: Thank you for your valuable feedback and for pointing out these relevant references. Given that there is a strict limit of 10 references for commentaries, we refrained from citing them, but in the revised version of the manuscript we mention that there is additional and more detailed literature on these topics already available and that these topics have been discussed within the scientific community.
